# Repetitive hypoglycemia reduces activation of glucose-responsive neurons in C1 and C3 medullary brain regions to subsequent hypoglycemia

**DOI:** 10.1152/ajpendo.00051.2019

**Published:** 2019-04-23

**Authors:** Zohra M. Kakall, Mary M. Kavurma, E. Myfanwy Cohen, Peter R. Howe, Polina E. Nedoboy, Paul M. Pilowsky

**Affiliations:** ^1^The Heart Research Institute, Newtown, New South Wales, Australia; ^2^Department of Physiology, Sydney Medical School, The University of Sydney, Sydney, New South Wales, Australia; ^3^Sydney Medical School, The University of Sydney, Sydney, New South Wales, Australia; ^4^School of Biomedical Sciences and Pharmacy, University of Newcastle and Institute for Resilient Regions, University of Southern Queensland, Springfield, Queensland, Australia

**Keywords:** catecholaminergic neurons, dorsomedial medulla, epinephrine, HAAF, ventrolateral medulla

## Abstract

The impaired ability of the autonomic nervous system to respond to hypoglycemia is termed “hypoglycemia-associated autonomic failure” (HAAF). This life-threatening phenomenon results from at least two recent episodes of hypoglycemia, but the pathology underpinning HAAF remains largely unknown. Although naloxone appears to improve hypoglycemia counterregulation under controlled conditions, hypoglycemia prevention remains the current mainstay therapy for HAAF. Epinephrine-synthesizing neurons in the rostroventrolateral (C1) and dorsomedial (C3) medulla project to the subset of sympathetic preganglionic neurons that regulate peripheral epinephrine release. Here we determined whether or not C1 and C3 neuronal activation is impaired in HAAF and whether or not 1 wk of hypoglycemia prevention or treatment with naloxone could restore C1 and C3 neuronal activation and improve HAAF. Twenty male Sprague-Dawley rats (250–300 g) were used. Plasma epinephrine levels were significantly increased after a single episode of hypoglycemia (*n* = 4; 5,438 ± 783 pg/ml vs. control 193 ± 27 pg/ml, *P* < 0.05). Repeated hypoglycemia significantly reduced the plasma epinephrine response to subsequent hypoglycemia (*n* = 4; 2,179 ± 220 pg/ml vs. 5,438 ± 783 pg/ml, *P* < 0.05). Activation of medullary C1 (*n* = 4; 50 ± 5% vs. control 3 ± 1%, *P* < 0.05) and C3 (*n* = 4; 45 ± 5% vs. control 4 ± 1%, *P* < 0.05) neurons was significantly increased after a single episode of hypoglycemia. Activation of C1 (*n* = 4; 12 ± 3%, *P* < 0.05) and C3 (*n* = 4; 19 ± 5%, *P* < 0.05) neurons was significantly reduced in the HAAF groups. Hypoglycemia prevention or treatment with naloxone did not restore the plasma epinephrine response or C1 and C3 neuronal activation. Thus repeated hypoglycemia reduced the activation of C1 and C3 neurons mediating adrenal medullary responses to subsequent bouts of hypoglycemia.

## INTRODUCTION

Type 1 diabetic (T1D) patients are at increased risk of developing life-threatening hypoglycemia due to the imperfect regulation of exogenously administered insulin. The sympathoadrenal response that restores blood glucose (BG) levels is weakened after a preceding episode of hypoglycemia. This eventually leads to a blunted counterregulatory response occurring at progressively lower BG levels. This phenomenon is termed “hypoglycemia-associated autonomic failure” (HAAF). Both the sympathoadrenal counterregulatory response and the behavioral adaptation to hypoglycemia are initiated at lower BG levels in HAAF. Together with reduced neurogenic and neuroglycopenic symptoms of hypoglycemia, HAAF significantly contributes to the development of hypoglycemia unawareness. Unawareness of hypoglycemia is a significant burden on the health care system worldwide, as well as a major cause of recurrent morbidity and mortality in patients with diabetes (5, 14, 62).

Glucagon and epinephrine are important counterregulatory hormones that are released after hypoglycemia in healthy humans. The epinephrine response following sympathetic neural activation is critical in T1D patients and is due to islet cell failure and the subsequent inability to increase glucagon levels after hypoglycemia (3, 15). Thus a better understanding of the pathophysiology underlying HAAF is critical to reducing the incidence of severe hypoglycemia by ensuring an adequate plasma epinephrine response. Current preventative approaches to reduce the incidence of severe hypoglycemia require the meticulous management of BG levels (11, 12). Clinical studies in which hypoglycemia is induced with a hyperinsulinemic-hypoglycemic clamp preparation show that coinfusion of naloxone, an opioid receptor antagonist, improves epinephrine release and endogenous glucose production in both healthy and T1D subjects (4, 27, 42, 57). Despite the use of hypoglycemia prevention and the promise of naloxone therapy, the mechanisms whereby epinephrine secretion and glucose recovery appear to be improved after these treatments remain unknown.

Hypoglycemia-induced activation of the sympathoadrenal axis results in the release of epinephrine from chromaffin cells (37). Adrenergic neurons of the rostroventrolateral (C1) and dorsomedial (C3) medulla oblongata contain axonal projections to adrenal sympathetic preganglionic neurons (31, 37, 40, 51). Furthermore, hypoglycemia- or glucoprivation-induced activation of C1 and C3 neurons results in epinephrine release from chromaffin cells located in the adrenal medulla (29, 37, 44, 45, 49). These findings suggest that after hypoglycemia a positive association exists between the activation state of medullary catecholaminergic (C1 and C3) neurons and downstream epinephrine secretion. Therefore, the improvements in epinephrine release following hypoglycemia prevention or naloxone therapy may be due to the restoration of C1 and C3 neuronal activity.

In the present study, we used a rodent model of HAAF (adapted from Ref. 48) to determine whether repeated insulin-induced hypoglycemia reduced the activation of C1 and C3 medullary neurons. Our specific aims were to determine whether *1*) a 3-day repeated hypoglycemia protocol reduces C1 and C3 neuronal activation and *2*) a hypoglycemia prevention protocol or *3*) injection of naloxone during antecedent hypoglycemia in HAAF rats can restore C1 and C3 neuronal activation and epinephrine release after subsequent hypoglycemia.

## METHODS

### 

#### Animal procedures.

All experiments were conducted in accordance with the Australian Code of Practice for the Care and Use of Animals for Scientific Purposes (New South Wales: Animal Research Act 1985), certified by the National Health and Medical Research Council of Australia. The protocol for this study was reviewed and approved by the Sydney Local Health District Animal Welfare Committee (protocol no. 2018/014). Unfasted male Sprague-Dawley rats (*n* = 20) weighing between 250 and 300 g were used in this study. Animals were fed ad libitum with a standard irradiated rat diet (Specialty Feeds) and autoclaved reverse-osmosis water.

#### Blood glucose analysis.

Rats were removed from their home cages on the morning of each experiment for the purpose of BG analysis and intraperitoneal drug injections. Rats were carefully placed in a restraint before BG levels were measured in order to prevent handling stress. BG levels were subsequently measured with a glucometer (Accu-Chek Performa) from a tail vein nick. If dilation of the tail vein was required to enhance vessel visibility, the tail was briefly placed in a warm water bath (~30°C) to enhance blood flow to the required area. Rats were removed from the restraint immediately afterward and carefully prepared for intraperitoneal injections. BG levels were measured once before drug administration and finally 2 h after injection.

#### Animal experiments.

**Fig. 1.**
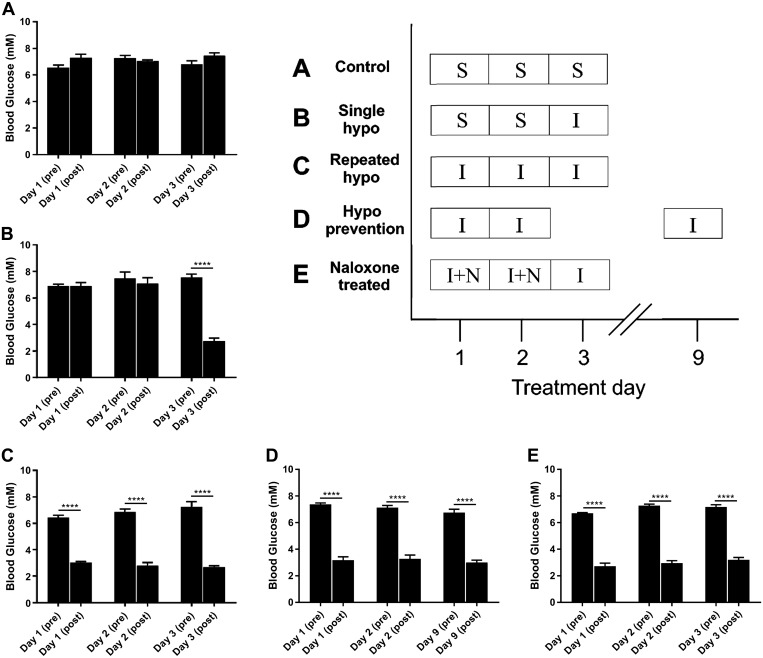
Insulin-induced hypoglycemia protocols (*inset*) and blood glucose levels in all treatment groups: grouped data (*n* = 4 animals/group) indicating blood glucose (BG) levels in groups treated with insulin (I; 5 U/kg) or saline control (S). BG levels were measured before injections and 2 h after injections. Hypoglycemia was achieved if BG ≤ 3.9 mM. In the control group (*A*), animals were injected with volume-matched 0.9% saline once daily for 3 days. In the single-hypoglycemia (hypo) group (*B*), saline was injected on *days 1* and *2*, followed by insulin on *day 3*. In the repeated-hypo group (*C*), insulin was injected on all days. In the hypo prevention group (*D*), insulin was injected on *days 1*, *2*, and *9*. In the naloxone (N)-treated group (*E*), insulin was injected on *days 1*, *2*, and *3*, followed by intraperitoneal injection of naloxone (1 mg/kg) after the 2-h BG levels were recorded on *days 1* and *2*. Data are means ± SE. Statistical significance was determined by 1-way ANOVA and multiple-comparison analysis with a Holm-Šidák correction. ****P < 0.0001, significantly different from all other groups.

Figure 1 provides a detailed description of the protocols. Rats were separated into groups receiving intraperitoneal injections of saline, insulin (5 U/kg), and/or naloxone (1 mg/kg). Injections were performed once daily over 3 days in each animal (*n* = 4 animals/group). The injection groups were as follows: control (volume-matched saline injections once daily for 3 days), single hypoglycemia (saline *days 1* and *2*, insulin *day 3*), repeated hypoglycemia-HAAF (insulin *days 1*, *2*, and *3*), hypoglycemia prevention (insulin *days 1*, *2*, and *9*), and naloxone treated (insulin followed by naloxone injection after the final glucose measurement 2 h after injection on *days 1* and *2*; only insulin was injected on *day 3*).

On the final day, after the BG measurement at 2 h, rats were injected with a lethal dose of pentobarbital sodium (1 ml of 65 mg/kg). Cardiac blood was collected from the left ventricle and stored in an EDTA tube. Rats were then transcardially perfused with 400 ml of phosphate-buffered saline (PBS; 10 mM sodium phosphate buffer in 0.9% sodium chloride at pH 7.4) followed by 400 ml of 10% neutral buffered formalin (Sigma-Aldrich, Darmstadt, Germany). Brain tissue was postfixed at room temperature for 24 h, placed in PBS treated with merthiolate, and stored at 4°C until required. Brain stems were separated and cut into 40-μm coronal sections with a vibrating microtome (Leica VT1200). Sections were collected sequentially into five individual pots containing cryoprotectant and stored at −20°C until required for immunohistochemistry.

#### Epinephrine ELISA.

Blood samples were centrifuged down at a speed of 4,750 rpm at 4°C for 10 min, and the plasma was collected and stored at −80°C for biochemical analysis. Fifty-microliter samples were used from each group, and plasma epinephrine concentrations were measured with a BA E-5100 Adrenaline Research ELISA kit (Taylor Biomedical).

#### Immunohistochemical analysis of Fos and phenylethanolamine-N-methyltransferase.

Before the addition of antibodies, brain stem sections were washed (3 × 30 min) with PBS containing 0.3% Triton X. Sections were incubated with 100 µl of donkey serum (Sigma-Aldrich, catalog no. D9663-10ML), 900 µl of PBS-Tris buffer containing 0.1% merthiolate and 0.3% Triton X-100, and 1 µl of the primary antibodies phenylethanolamine-*N*-methyltransferase (PNMT, 1:1,000 dilution, rabbit polyclonal; produced by P. R. Howe) and Fos (1:1,000 dilution, guinea pig polyclonal; Synaptic Systems, catalog. no. 226004) for 72 h at 4°C on an orbital shaker. After this, sections were once again washed (3 × 30 min) with PBS before the addition of secondary antibodies. The PNMT antibody was validated for specificity by P. R. Howe (20). This was followed by the addition of 980 µl of PBS-Tris buffer containing 0.1% merthiolate, 20 µl of donkey serum, and 4 µl of the secondary antibodies anti-rabbit Cy3 (1:500 dilution; Jackson Immunoresearch, catalog no. 711-166-152) for the detection of PNMT and anti-guinea pig Alexa Fluor 488 (1:500 dilution; Jackson Immunoresearch, catalog no. 706-546148) for the detection of Fos. All sections were protected from light and placed on an orbital shaker at room temperature for 24 h. Finally, the sections were washed once again with PBS (3 × 30 min) and mounted on glass slides. Mounted sections were allowed to dry before being coverslipped with ProLong Diamond antifade mountant (ThermoFisher Scientific) and sealed with clear nail polish.

#### Microscopy.

Immunofluorescent (×20 magnification) images for the cell count analysis were captured with a Zeiss Axio Scan Z1 slide scanner microscope. Exposure time settings remained consistent for all sections at the time of imaging. Contrast and brightness of the resulting images were adjusted to maximize our ability to count cells with minimal background interference. Slides were coded during microscopy, and all analysis was performed under “blinded” conditions to avoid experimenter bias. For illustrative purposes, photomicrographs (×40 magnification) were obtained with a Zeiss Axio Imager Z2.

#### Data analysis and statistics.

The total numbers of PNMT+ and double-stained Fos+PNMT+ cells in the C1 (bregma level −12.48 to −12.24) and C3 (bregma level −12.48 to −12.24) regions were quantified with Zen 2.3 (Blue edition). A rectangular region of interest was placed bilaterally in the C1 region (5,500 × 3,700 µm) and in the C3 region (7,000 × 4,000 µm). In each treatment group (*n* = 4 animals/group), the total numbers of cells in two sections per region from each animal were recorded. The average number in each group was used to calculate the total number of activated C1 and C3 neurons(expressed as % Fos+PNMT+/PNMT+). Comparisons were made with one- or two-way ANOVA as appropriate. Statistical analysis was performed with GraphPad Prism software (v7.02). Multiple comparisons were made with the post hoc Holm-Šidák test where appropriate.

## RESULTS

### 

#### Insulin-induced hypoglycemia protocols.

Intraperitoneal injections of insulin (5 U/kg) were administered to induce hypoglycemia once daily for 3 days in the HAAF treatment groups (Fig. 1). Insulin was injected on *day 3* only in the single-hypoglycemia group. Control rats were provided with an intraperitoneal injection of volume-matched saline. The threshold for hypoglycemia was BG ≤ 3.9 mM. The saline-treated control group (*n* = 4) remained normoglycemic on all 3 days (Fig. 1*A*). In the single-hypoglycemia group (*n* = 4), BG levels after saline injections on *days 1* and *2* remained at normoglycemia, whereas insulin significantly reduced BG levels from 7.5 ± 0.3 mM to 2.8 ± 0.2 mM on *day 3* (Fig. 1*B*). In the repeated-hypoglycemia group (*n* = 4), BG levels 2 h after injection of insulin were significantly reduced from the matched preinjection time points (Fig. 1*C*): 3.1 ± 0.1 mM (*day 1*), 2.8 ± 0.2 mM (*day 2*), and 2.7 ± 0.1 mM (*day 3*). In the 1-wk hypoglycemia prevention group (*n* = 4), BG levels after insulin were significantly reduced to 3.2 ± 0.3 mM (*day 1*), 3.3 ± 0.3 mM (*day 2*), and 3.0 ± 0.2 mM (*day 9*) (Fig. 1*D*). In the naloxone-treated group (*n* = 4), BG levels after insulin were significantly reduced to 2.8 ± 0.2 mM (*day 1*), 3.0 ± 0.2 mM (*day 2*), and 3.2 ± 0.2 mM (*day 3*) (Fig. 1*E*).

#### Antecedent insulin-induced hypoglycemia reduces plasma epinephrine levels after subsequent hypoglycemia.

**Fig. 2.**
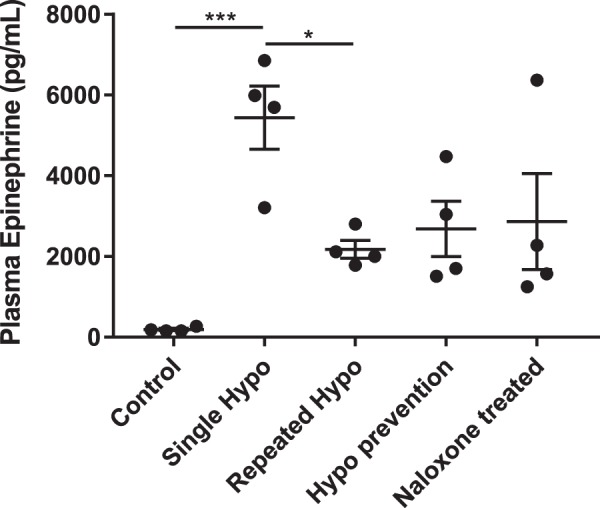
Antecedent insulin-induced hypoglycemia reduces plasma epinephrine levels after subsequent hypoglycemia: grouped data indicating plasma epinephrine levels measured from blood samples collected on the final day of injections from each animal. Plasma epinephrine levels were not affected in saline-treated control rats (*n* = 4). In the single-hypoglycemia (hypo) group, 1 episode of hypoglycemia significantly increased plasma epinephrine levels (*n* = 4). In the repeated-hypo group, plasma epinephrine levels after the last episode of hypoglycemia were significantly reduced compared with the single-hypo group (*n* = 4). Plasma epinephrine levels remained at levels similar to the repeated-hypo group after 1 wk of hypoglycemia prevention (*n* = 4) or naloxone treatment (*n* = 4). Data are means ± SE. Statistical significance was determined by 1-way ANOVA and multiple-comparison analysis with a Holm-Šidák correction. *P < 0.05, ***P < 0.001, significantly different from all other groups.

Plasma epinephrine levels were measured by ELISA to determine how the sympathoadrenal response to hypoglycemia was affected in each treatment group (Fig. 2). Blood was collected by cardiac puncture 2 h after injection on the final day of treatment in all animals. Plasma epinephrine levels significantly increased in the single-hypoglycemia group compared with control (5,438 ± 783 pg/ml vs. control 193 ± 27 pg/ml; Fig. 2). Repeated hypoglycemia significantly reduced the plasma epinephrine response to subsequent hypoglycemia (2,179 ± 220 pg/ml vs. single hypoglycemia 5,438 ± 783 pg/ml; Fig. 2). In the HAAF rats, hypoglycemia prevention or naloxone treatment failed to restore the plasma epinephrine response to subsequent hypoglycemia.

#### Antecedent insulin-induced hypoglycemia reduces activation of adrenergic neurons in medullary C1 and C3 nuclei after subsequent hypoglycemia.

**Fig. 3.**
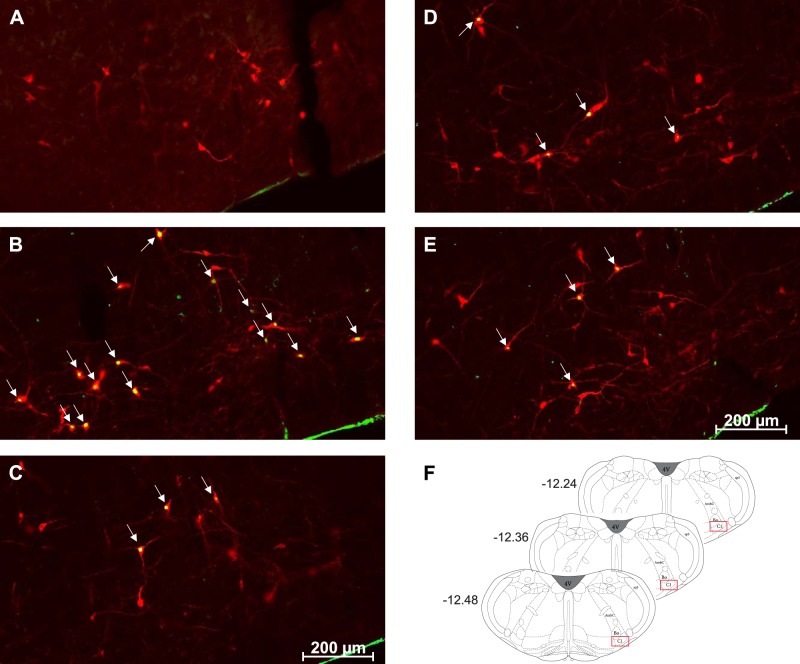
Representative images (×20) of treated C1 sections depicting Fos and phenylethanolamine-*N*-methyltransferase (PNMT) immunolabeling. *A–E*: representative photomicrographs (×20 magnification) of Fos (green) and PNMT (red) immunolabeling in the C1 medullary region of rat tissue (*n* = 4 animals/group). Cy3 fluorescence is pseudocolored red, and Alexa Fluor 488 is represented in green. Images represent staining in control (*A*), single-hypoglycemia (hypo) (*B*), repeated-hypo (*C*), hypo prevention (*D*), and naloxone-treated (*E*) animal tissue. Overlapping Fos+PNMT+ cells are indicated by arrows. *F*: bregma levels used for analysis. AmbC, ambiguus nucleus, compact part; Bo, Bötzinger complex; Sp5, spinal trigeminal nucleus; 4V, fourth ventricle.

**Fig. 4.**
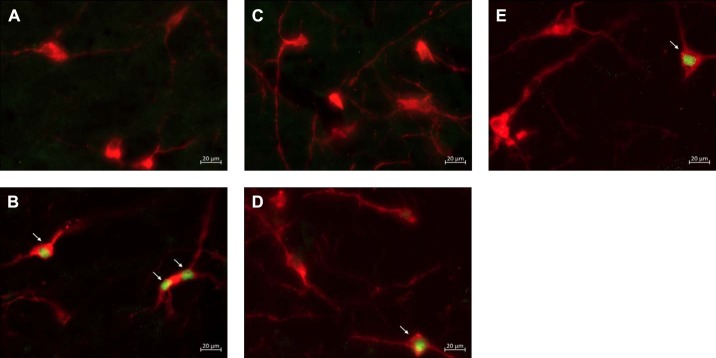
Representative images (×40) of treated C1 sections depicting Fos and phenylethanolamine-*N*-methyltransferase (PNMT) immunolabeling: representative photomicrographs (×40 magnification) of Fos (green) and PNMT (red) immunolabeling in the C1 medullary region of rat tissue (*n* = 4 animals/group). Cy3 fluorescence is pseudocolored red, and Alexa Fluor 488 is represented in green. Images represent staining in control (*A*), single-hypoglycemia (hypo) (*B*), repeated-hypo (*C*), hypo prevention (*D*), and naloxone-treated (*E*) animal tissue. Overlapping Fos+PNMT+ cells are indicated by arrows.

**Fig. 5.**
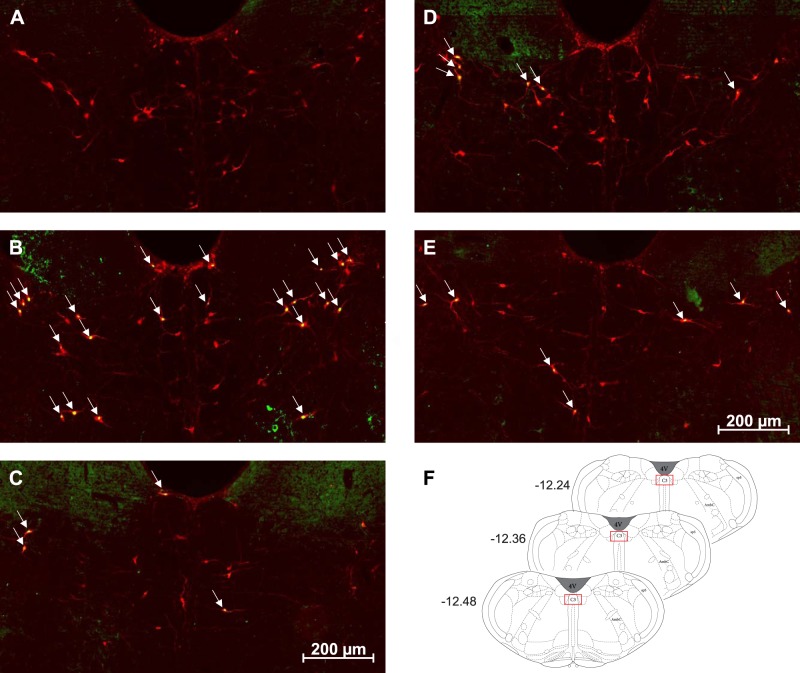
Representative images (×20) of treated C3 sections depicting Fos and phenylethanolamine-*N*-methyltransferase (PNMT) immunolabeling. *A–E*: representative photomicrographs (×20 magnification) of Fos (green) and PNMT (red) immunolabeling in the C3 medullary region of rat tissue (*n* = 4 animals/group). Cy3 fluorescence is pseudocolored red, and Alexa Fluor 488 is represented in green. Images represent staining in control (*A*), single-hypoglycemia (hypo) (*B*), repeated-hypo (*C*), hypo prevention (*D*), and naloxone-treated (*E*) animal tissue. Overlapping Fos+PNMT+ cells are indicated by arrows. *F*: bregma levels used for analysis. AmbC, ambiguus nucleus, compact part; Bo, Bötzinger complex; Sp5, spinal trigeminal nucleus; 4V, fourth ventricle.

**Fig. 6.**
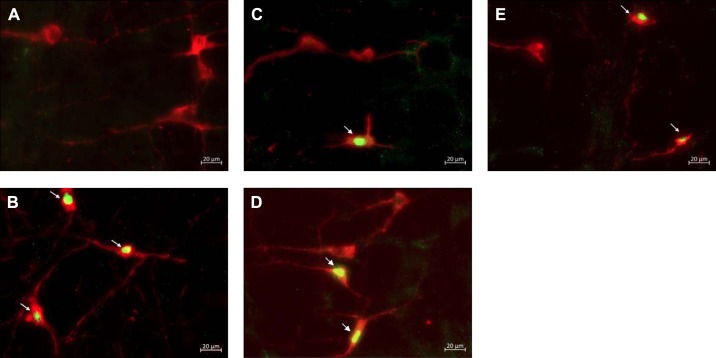
Representative images (×40) of treated C3 sections depicting Fos and phenylethanolamine-*N*-methyltransferase (PNMT) immunolabeling: representative photomicrographs (×40 magnification) of Fos and PNMT immunolabeling in the C3 medullary region of rat tissue (*n* = 4 animals/group). Cy3 fluorescence is pseudocolored red, and Alexa Fluor 488 is represented in green. Images represent staining in control (*A*), single-hypoglycemia (hypo) (*B*), repeated-hypo (*C*), hypo prevention (*D*), and naloxone-treated (*E*) animal tissue. Overlapping Fos+PNMT+ cells are indicated by arrows.

**Fig. 7.**
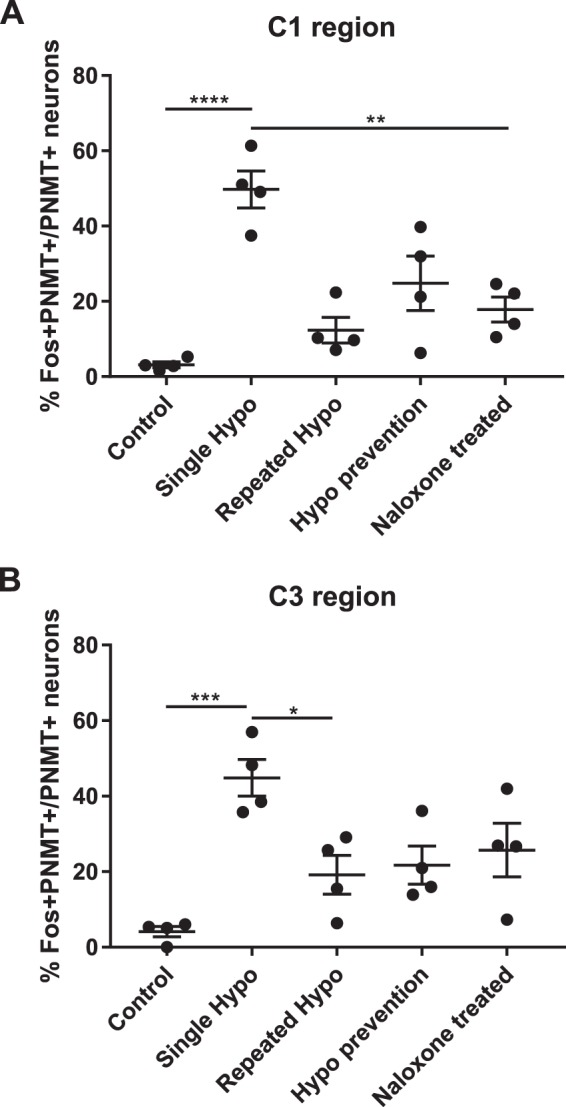
Antecedent insulin-induced hypoglycemia reduces activation of adrenergic neurons in medullary C1 and C3 nuclei after subsequent hypoglycemia: neuronal activation represented by double-stained Fos+ phenylethanolamine-*N*-methyltransferase (PNMT)+ neurons expressed as % of total PNMT+ neurons in the C1 (*A*) and C3 (*B*) regions (*n* = 4 animals/group). The ratio of activated catecholaminergic neurons in both regions was significantly higher in the single-hypoglycemia (hypo) group compared with the repeated-hypo group. There was no recovery in C1 or C3 neuronal activation after hypo prevention or naloxone treatment. Data are means ± SE. Statistical significance was determined by 1-way ANOVA and multiple-comparison analysis with a Holm-Šidák correction. *P < 0.05, **P < 0.01, ***P < 0.001, ****P < 0.0001, significantly different from all other groups.

The total number of Fos and PNMT dually immunoreactive neurons in the C1 and C3 medullary regions was quantified in all treatment groups (Figs. 3–6). In the C1 region, repeated saline injections did not significantly increase Fos expression (Fig 3*A*, Fig. 4*A*, and Fig. 7*A*). A single episode of hypoglycemia significantly increased the % Fos+PNMT+/PNMT+ neurons compared with all other treatment groups (50 ± 5% vs. control 3 ± 1%; Fig. 3*B*, Fig. 4*B*, and Fig. 7*A*). Furthermore, repeated hypoglycemia significantly reduced the activation of these neurons after subsequent hypoglycemia (12 ± 3% vs. single hypoglycemia 50 ± 5%; Fig. 3*C*, Fig. 4*C*, and Fig. 7*A*). Hypoglycemia prevention (Fig. 3*D*, Fig. 4*D*, and Fig. 7*A*) or naloxone treatment (Fig. 3*E*, Fig. 4*E*, and Fig. 7*A*) did not restore C1 neuronal activation.

In the C3 region, repeated saline injections did not significantly increase Fos expression (Fig. 5*A*, Fig. 6*A*, and Fig. 7*B*). A single episode of hypoglycemia significantly increased the % Fos+PNMT+/PNMT+ neurons compared with saline control (45 ± 5% vs. control 4 ± 1%; Fig. 5*B*, Fig. 6*B*, and Fig. 7*B*). Repeated hypoglycemia significantly reduced the activation of adrenergic C3 neurons after subsequent hypoglycemia (19 ± 5% vs. single hypoglycemia 45 ± 5%; Fig. 5*C*, Fig. 6*C*, and Fig. 7*B*). Hypoglycemia prevention (Fig. 5*D*, Fig. 6*D*, and Fig. 7*B*) or naloxone treatment (Fig. 5*E*, Fig. 6*E*, and Fig. 7*B*) did not restore C3 neuronal activation.

**Fig. 8.**
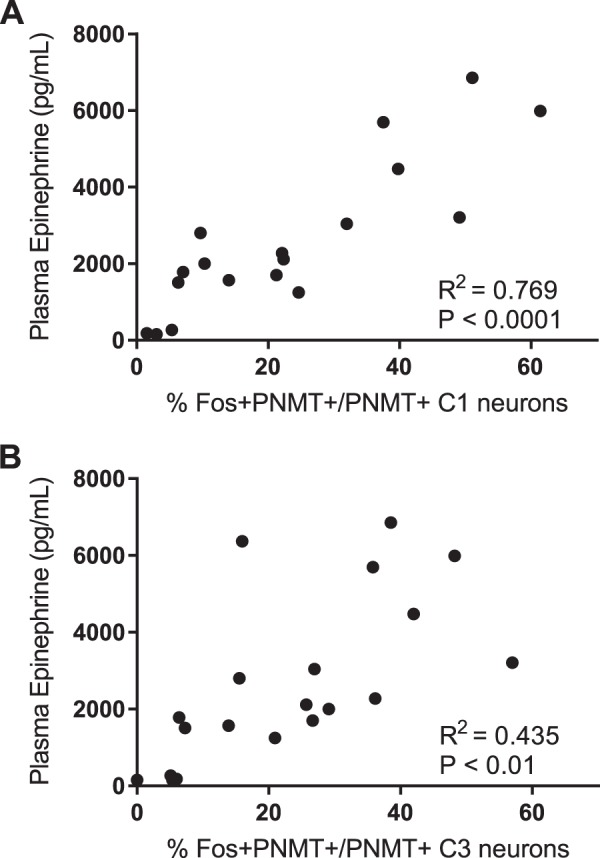
Correlation between plasma epinephrine levels and C1 and C3 neuronal activity: positive correlation between plasma epinephrine levels and the degree of C1 (*A*) and C3 (*B*) neuronal activation after hypoglycemia. Statistical significance was determined by a Pearson correlation test with GraphPad Prism software. PNMT, phenylethanolamine-*N*-methyltransferase.

A direct correlation between plasma epinephrine levels and C1 or C3 neuronal activation was observed (Fig. 8), suggesting that increased C1 (Fig. 8*A*; *R*^2^ = 0.7692) and C3 (Fig. 8*B*; *R*^2^ = 0.4352) neuronal activation is closely correlated with increasing plasma epinephrine levels.

#### Repeated hypoglycemia does not alter neuronal PNMT expression in medullary C1 and C3 regions.

**Fig. 9.**
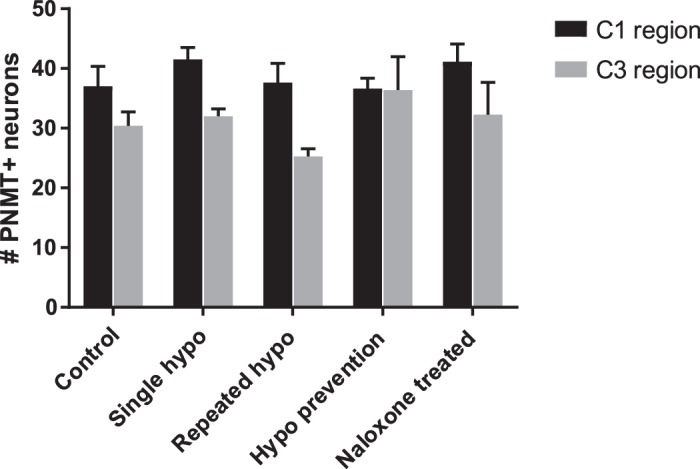
Repeated hypoglycemia (hypo) does not alter neuronal phenylethanolamine-*N*-methyltransferase (PNMT) expression in the medullary C1 and C3 regions: neuronal cell counts depicting the total number of PNMT+ neurons in both the C1 and C3 regions (*n* = 4 animals/group). The total number of adrenergic neurons in the C1 and C3 regions did not vary between groups. Data are means ± SE. Statistical significance was determined by 2-way ANOVA. *P* > 0.05, not significantly different from all other groups.

The total numbers of PNMT+ neurons in the C1 and C3 regions were quantified. These values were not significantly different across all treatment groups (Fig. 9).

## DISCUSSION

The novel finding of the present study is that in HAAF activation of C1 and C3 glucose-responsive neurons is reduced after two episodes of antecedent hypoglycemia. Plasma epinephrine release and the sympathoadrenal response to hypoglycemia were also reduced in HAAF rats. Interestingly, the hypoglycemia prevention protocol (Fig. 1) and injection of naloxone during antecedent hypoglycemia had little effect on the activity of C1 and C3 neurons. Only 50% of the expected downstream sympathoadrenal response to hypoglycemia was recovered, which was comparable to the repeated-hypoglycemia-HAAF treatment group (see Fig. 2).

Hypoglycemia initiates the activation of hypothalamic and medullary pathways that enhance pancreatic glucagon and adrenal medullary epinephrine secretion (24, 25, 58–60). Recently, Senthilkumaran et al. (49) used a diaminobenzidine staining method to demonstrate the presence of glucose-responsive neurons in the C1 and C3 medullary regions as well as in the chromaffin cells of the adrenal medulla. Similar diaminobenzidine staining methodology revealed that Fos expression is also present in the C1 and C3 regions after systemic 2-deoxyglucose (2-DG)-induced glucoprivation (41, 45). Repeated 2-DG-induced glucoprivation significantly reduced Fos expression in the C1 and C3 medullary regions (47). Plasma epinephrine levels were not reported in the study by Sanders and Ritter (47), but the hyperglycemic effect of 2-DG was abolished in the repeated 2-DG-treated group. It is worth noting that 2-DG is a glucose analog that competes for glucose uptake and is therefore a potent inhibitor of glycolysis. The systemic administration of 2-DG produces hyperglycemia and initiates the sympathoadrenal counterregulatory response causing both epinephrine and glucagon to be released peripherally (21–24). Although a similar subset of medullary neurons is activated after either 2-DG or insulin injections, the physiological mechanisms underlying HAAF must be determined with models of insulin-induced hypoglycemia, rather than glucoprivation, to maintain biological relevance. The present study is the first to link reduced activation of C1 and C3 medullary neurons in a rodent model of insulin-induced HAAF. We believe the activation of C1 and C3 neurons is required for downstream epinephrine release, and any treatments that serve to improve activation of these medullary neurons will subsequently restore the downstream sympathoadrenal response (Fig. 8). The central pathway resulting in downstream activation of C1 and C3 neurons after hypoglycemia remains unknown, but numerous studies suggest that the activation of glucose-inhibited neurons in the hypothalamic ventromedial nucleus and perifornical nucleus may trigger the counterregulatory response to hypoglycemia (24, 25, 46, 53, 59, 63).

Intensive insulin therapy enables a profound reduction in microvascular complications (neuropathy, retinopathy, and nephropathy) commonly caused by T1D (8, 64). The imperfect pharmacokinetic and pharmacodynamic regulation of insulin requires careful consideration when administering the drug for treatment. A single episode of antecedent hypoglycemia can reduce the sympathoadrenal response to subsequent hypoglycemia, with repeated episodes of hypoglycemia leading to the development of HAAF (50). Established HAAF, together with impaired awareness of hypoglycemia, increases the risk of developing severe hypoglycemia, potentially leading to coma or death (6, 7). Improvement in glycemic control is achieved clinically by regular monitoring of blood glucose levels together with structured education programs aiming to reduce the incidence of mild and severe hypoglycemic events (9, 19). Here we tested the hypothesis that hypoglycemia prevention would restore the sympathoadrenal response to hypoglycemia as well as C1 and C3 neuronal activation in our model of HAAF. Our rationale was based on clinical findings indicating that both the symptomatic and epinephrine responses were restored by ~50% in T1D patients after 2 wk of meticulous glycemic management (11). Despite the healthy Sprague-Dawley background of our HAAF rats, delayed induction of the final hypoglycemia by 1 wk was insufficient to restore an adequate neuronal, or epinephrine, response to subsequent hypoglycemia.

Naloxone is a rapidly acting opioid antagonist that crosses the blood-brain barrier and is commonly used to reverse opioid-induced overdose. It is a nonselective competitive antagonist at all central and peripherally located opioid receptors, with a particularly high affinity for the µ-receptor subtype. Coinfusion of insulin and naloxone during antecedent hypoglycemia in human studies improved the epinephrine response, endogenous glucose production, and hypoglycemia symptom scores after subsequent hypoglycemia (27, 32, 42, 57). Notwithstanding clinical studies demonstrating the potential of naloxone in improving the sympathoadrenal and symptomatic response to hypoglycemia, the exact mechanisms mediating this effect remain unknown. Conflicting findings in rodent models of HAAF indicate that intraperitoneal injection of 5 mg/kg naloxone 15 min before 10 U/kg insulin injections did not restore plasma epinephrine levels after subsequent hypoglycemia (48). Our measurements of plasma epinephrine are comparable to those of Senthilkumaran and Bobrovskaya (48) despite the fact that we lowered the dose of naloxone to 1 mg/kg to prevent any potential nonspecific effects (13). We also injected naloxone 2 h after insulin-induced hypoglycemia to determine whether a more physiologically relevant time frame of administration would recover the epinephrine response. Coadministration of naloxone and insulin is yet to be tested in animal models of HAAF, as the ideal option would be to select a drug that would be effective for use during established hypoglycemia.

In a controlled clinical study by Leu et al. (27), the epinephrine response in nondiabetic humans was reduced after recurrent hypoglycemia, although these individuals possessed an intact glucagon response. Furthermore, the epinephrine response was successfully restored in these healthy subjects with continuous naloxone infusions, despite their ability to mount a sufficient glucagon response. On the other hand, the glucagon response to hypoglycemia is completely lost in T1D patients (57), and after recurrent hypoglycemia the impaired plasma epinephrine response is improved with naloxone infusions. In light of this evidence, although measurements of plasma glucagon would be informative, we do not believe an intact glucagon response would have affected the ability of naloxone to restore the epinephrine response.

Central activation of neurons in the medulla oblongata may regulate improvements in adrenal medullary chromaffin cell catecholamine synthesis and secretion. Although we did not quantify adrenal medullary chromaffin cell responsiveness, earlier studies have shown that naloxone, but not methylnaltrexone (another broad-spectrum opioid antagonist that does not cross the blood-brain barrier), significantly improves the levels of tyrosine hydroxylase phosphorylation of Ser31 and Ser40 in the adrenal gland of HAAF rats (48). Together, these results indicate that the pathway increasing tyrosine hydroxylase phosphorylation in the adrenal gland after HAAF may be mediated by slight improvements in central neuronal responsiveness following naloxone treatment that remain undetected by our method of assessment. The improvement we observed was not significant and did not restore plasma epinephrine release in the present study.

The development of a peripheral defect at the level of epinephrine-secreting chromaffin cells after recurrent hypoglycemia contributes to the impaired counterregulatory secretion of epinephrine (28, 38). Ma et al. (28) showed that in a rodent model of HAAF defects existed in the adrenal gland but not in the downstream hepatic components of the counterregulatory response. These results suggest that impaired sympathetic efferent neuronal signaling causes reduced activation of epinephrine-secreting cells in the adrenal gland, since cell death was not observed in the adrenal gland (28). Although their results did not reach significance, Sivitz et al. (52) showed that adrenal sympathetic nerve activity was reduced in HAAF rats exposed to subsequent hypoglycemia. These observations do not exclude the possibility that there is also impaired activation of C1 and C3 neurons after repeated hypoglycemia. The question of the importance of C1 and C3 neuronal activation in restoring the counterregulatory response to hypoglycemia in HAAF remains unanswered by the present study. Additional approaches targeting restoration of C1 and C3 neuronal activation, including optogenetic activation of C1 and/or C3 neurons and longer hypoglycemia avoidance time frames, are required to clarify the relative importance of C1 and C3 neurons as potential therapeutic targets for improving the counterregulatory epinephrine response in HAAF. Without data showing that the restoration of C1 and/or C3 neuronal activation also restores the sympatho-adrenomedullary response, the defective counterregulatory response to repeated hypoglycemia could potentially be due to changes or adaptations occurring in other brain regions, such as those located in the ventromedial nucleus, arcuate nucleus, and the lateral hypothalamus.

The mechanism whereby hypoglycemia prevention restores the sympathoadrenal response in HAAF remains unknown (11). The present study tested two treatments for HAAF to determine the physiological underpinnings that appear to reverse the phenomenon. The reversibility of HAAF by hypoglycemia prevention bears resemblance to the detoxification processes used to reverse opioid dependence (26, 55). Shifting of the hypoglycemia counterregulatory response to occur at lower BG levels can also be likened to the development of hypoglycemia tolerance. β-Endorphin is a highly selective endogenous agonist of µ-opioid receptors, and its levels are elevated in both plasma and cerebrospinal fluid after hypoglycemia (39, 43). Both C1 and non-C1 neurons in the ventrolateral medulla contain µ-opioid receptor-immunoreactive synaptic inputs that reduce neuronal efferent signaling upon activation (1, 2, 17, 18, 33, 36). Recently, we also showed that the selective activation of µ-opioid receptors in the rostral ventrolateral medulla attenuates adrenal sympathetic outflow by ~50% and completely blocks the counterregulatory response to glucoprivation (22). To the best of our knowledge, it remains unknown whether C3 neurons also contain µ-opioid receptor-immunoreactive synaptic inputs. Activation of µ-opioid receptors results in protein kinase C-mediated activation of *N*-methyl-d-aspartate (NMDA) receptors, which are implicated in tolerance, dependence, and inhibition of neural pathways (55). Furthermore, the NMDA receptor antagonist MK-801 inhibits the development of tolerance to the analgesic properties of morphine (30, 55, 56). These data suggest that the complete reversibility of HAAF after 3 mo may be due to an NMDA receptor-dependent adaptive alteration in the central pathways following µ-opioid receptor activation during hypoglycemia (11).

Although it would be of interest to determine whether or not Fos expression in the C1 cells is uniformly reduced after repeated bouts of hypoglycemia, the tissue analyzed in the present study was from the rostral portion of the C1 column, from bregma level −12.48 mm to −12.24 mm. The C1 cell column extends from bregma level −13.72 mm to −11.4 mm, with the bulbospinal component of the rostral C1 cell column residing between −13.0 mm and −11.4 mm (16). The rostral C1 neurons that selectively target the intermediolateral cell column of the spinal cord were of particular interest to us given that this proportion of C1 cells activate adrenal sympathetic preganglionic neurons, regulating downstream epinephrine release (34, 35, 44, 45). This does not discount the possibility that caudal C1 neurons projecting to the medial hypothalamus (paraventricular and arcuate nucleus) are also contributing to the downstream epinephrine response; however, the activation of caudal C1 cells is more closely linked to behavioral responses such as feeding following insulin-induced hypoglycemia (10, 44, 54, 61). Furthermore, Senthilkumaran et al. (49) showed that both caudal and rostral C1 cells are activated after insulin-induced hypoglycemia, so it would not be surprising if repeated hypoglycemia also reduced the activation of caudal C1 neurons, since the behavioral response to hypoglycemia (i.e., feeding) is reduced in HAAF (47). The present study falls short in providing an answer for what the relative contribution of caudal C1 neuronal activation is in terms of hypoglycemia-induced epinephrine secretion.

### 

#### Technical limitations.

In hypoglycemia, common signs and symptoms in humans include hunger, shakiness, tachycardia, dizziness, and sweatiness. We were unable to determine whether or not there were any of these changes in our conscious rodent model of HAAF. These findings highlight a key limitation in using this approach for modeling HAAF and awareness of hypoglycemia. Nonetheless, studying physiological mechanisms in animals is an invaluable resource that allows for a deeper understanding of the neuroendocrine mechanisms that define HAAF. Future studies should direct attention toward inducing HAAF in various rodent models of insulin-dependent diabetes mellitus (streptozotocin-induced T1D and obese Zucker). Longer hypoglycemia prevention protocols should also be considered despite the healthy/nondiabetic background of the animals.

In conclusion, the present study utilized immunohistochemical and biochemical techniques to show that the activation of C1 and C3 medullary neurons is impaired in a rodent model of HAAF. These reductions in neuronal activation contribute to the attenuated plasma epinephrine release in HAAF. Neither 1 wk of hypoglycemia prevention nor the injection of naloxone during antecedent hypoglycemia restored the activation of C1 and C3 neurons or the plasma epinephrine response. The present study highlights that the responsiveness of glucoregulatory C1 and C3 neurons is critical and must be considered when testing suitable therapies for HAAF.

#### Perspectives and significance.

Sympathoadrenal responsiveness can be measured both subjectively (neuroglycopenic and neurogenic symptoms) and objectively (hormone release) in humans. However, to achieve a greater mechanistic understanding of HAAF, an integrative physiological approach in experimental models of HAAF is required. Our study is the first to report a direct relationship between reduced glucose responsiveness in ventrolateral and dorsomedial medullary neurons leading to sympathoadrenal failure in a rodent model of HAAF. Additional experiments modeling HAAF on a T1D background and successfully restoring the activation of glucose-responsive C1 and C3 neurons are required. This will determine the relevance of C1 and C3 neurons in restoring the impaired sympathoadrenal response to hypoglycemia in HAAF.

## GRANTS

Work in the authors’ laboratories was supported by funding from the National Health and Medical Research Council of Australia (Grants 1065485 and 1024489), The Heart Research Institute, and the University of Sydney. Z. M. Kakall is supported by a University of Sydney Postgraduate Award (SC0649).

## DISCLOSURES

No conflicts of interest, financial or otherwise, are declared by the authors.

## AUTHOR CONTRIBUTIONS

Z.M.K., M.M.K., and P.M.P. conceived and designed research; Z.M.K. performed experiments; Z.M.K., M.M.K., and P.M.P. analyzed data; Z.M.K., M.M.K., E.M.C., P.E.N., and P.M.P. interpreted results of experiments; Z.M.K. and P.M.P. prepared figures; Z.M.K., M.M.K., and P.M.P. drafted manuscript; Z.M.K., M.M.K., E.M.C., P.R.H., P.E.N., and P.M.P. edited and revised manuscript; Z.M.K., M.M.K., E.M.C., P.R.H., P.E.N., and P.M.P. approved final version of manuscript.
